# Aggressive primary gastric lymphoma (PGL) masquerading as hepatocellular cancer (HCC) in alcoholic cirrhosis

**DOI:** 10.1186/s12876-021-01691-y

**Published:** 2021-03-04

**Authors:** Moiz Ahmed, Ahmed Al-Khazraji, Umer Syed, Tasur Seen, Aaron Walfish

**Affiliations:** Division of Gastroenterology and Hepatology, Icahn School of Medicine At Mount Sinai (Elmhurst) Hospital, 79-01 Broadway, Elmhurst, NY 11373 USA

**Keywords:** Primary gastric lymphoma, Cirrhosis, Hepatocellular carcinoma HCC, Case report

## Abstract

**Background:**

The gastrointestinal tract is sa well-known site for extranodal Non-Hodgkin lymphomas, with the stomach is known to be the most common site on lymphoma, primary gastric lymphoma (PGL). The lymphoproliferative disorder rarely occurs in patients with cirrhosis. We report a unique case of metastatic PGL in a patient with cirrhosis.

**Case presentation:**

A middle-aged male with decompensated alcoholic cirrhosis presented with two weeks of epigastric abdominal pain, abdominal distension, and jaundice. Abdominal triple-phase CT scan was consistent with cirrhosis, ascites, and multiple new hypodense liver lesions classified as an intermediate probability for HCC based on the LI-RADS classification system (LI RADS 3). Due to the CT findings in the setting of cirrhosis, a provisional diagnosis of HCC was made. Upper endoscopy revealed new multiple umbilicated submucosal nodules in the gastric body. Biopsy and immunostaining consistent with high-grade B-cell lymphoma. Targeted liver biopsy with similar morphology and immunostaining profile consistent with metastatic primary gastric DLBCL.

**Conclusions:**

The case highlights the importance of recognizing metastatic PGL in patients with underlying cirrhosis to differentiate lymphoma from hepatocellular cancer. Targeted liver biopsies with lymphoma immunostaining are required to make a diagnosis.

## Background

The gastrointestinal (GI) tract is a well-known site for extra nodal Non-Hodgkin lymphomas, representing 30–40% of all extra nodal lymphomas. The stomach is known to be the most common site followed by the small intestine and the colorectum. Potential risk factors for extra nodal Non-Hodgkin lymphomas include infections such as H. pylori, HIV, Hepatitis B, HTLV, and noninfectious diseases such as celiac disease, IBD, SLE, and immunosuppression [1]. The development of chronic liver disease and cirrhosis in patients with the pre-existing lymphoproliferative disorder has been well described. However, the development of lymphoma in patients with cirrhosis is extremely rare. This carries a diagnostic challenge and creates confusion among many clinicians that can be mistakenly diagnosed as HCC. We describe a unique and rare case of metastatic primary gastric lymphoma in patients with alcoholic cirrhosis.

## Case presentation

**Fig. 1:**
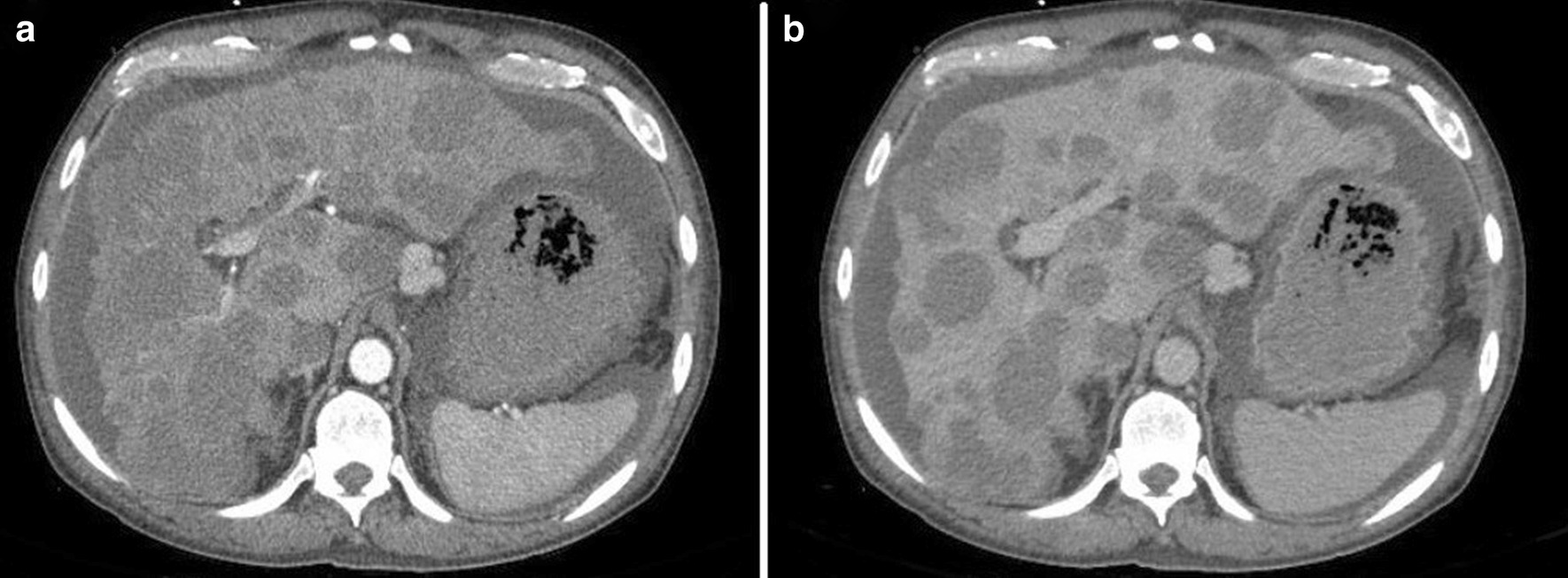
Cross sectional imaging CT scan abdomen demonstrates multiple hypodense hepatic lesions, **a** arterial phase. **b** Portal venous phase

**Fig. 2:**
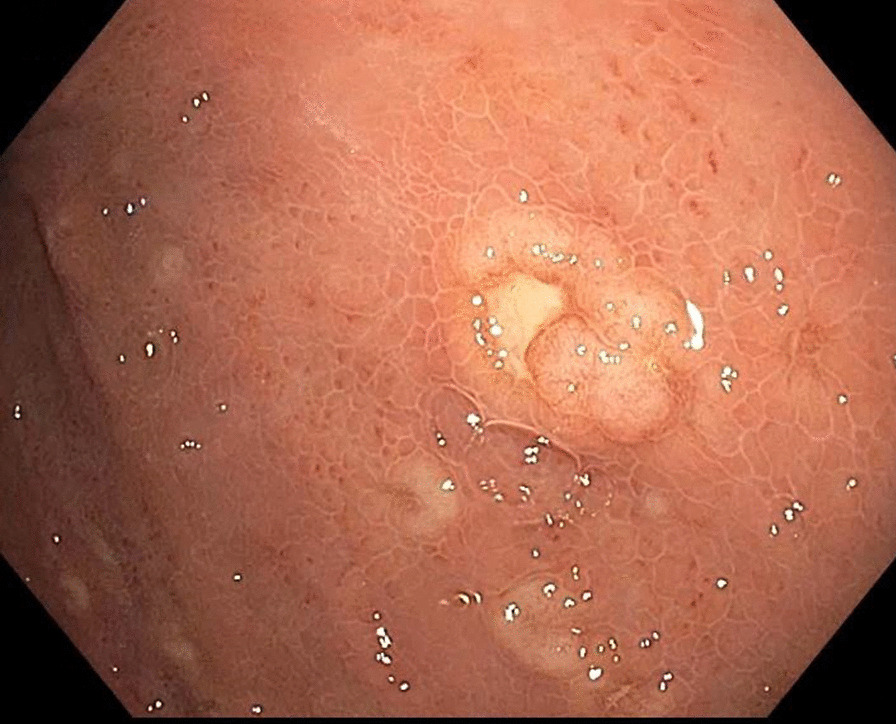
Endoscopy (EGD) image demonstrates multiple umbilicated, ulcerated lesions throughout the gastric body

**Fig. 3:**
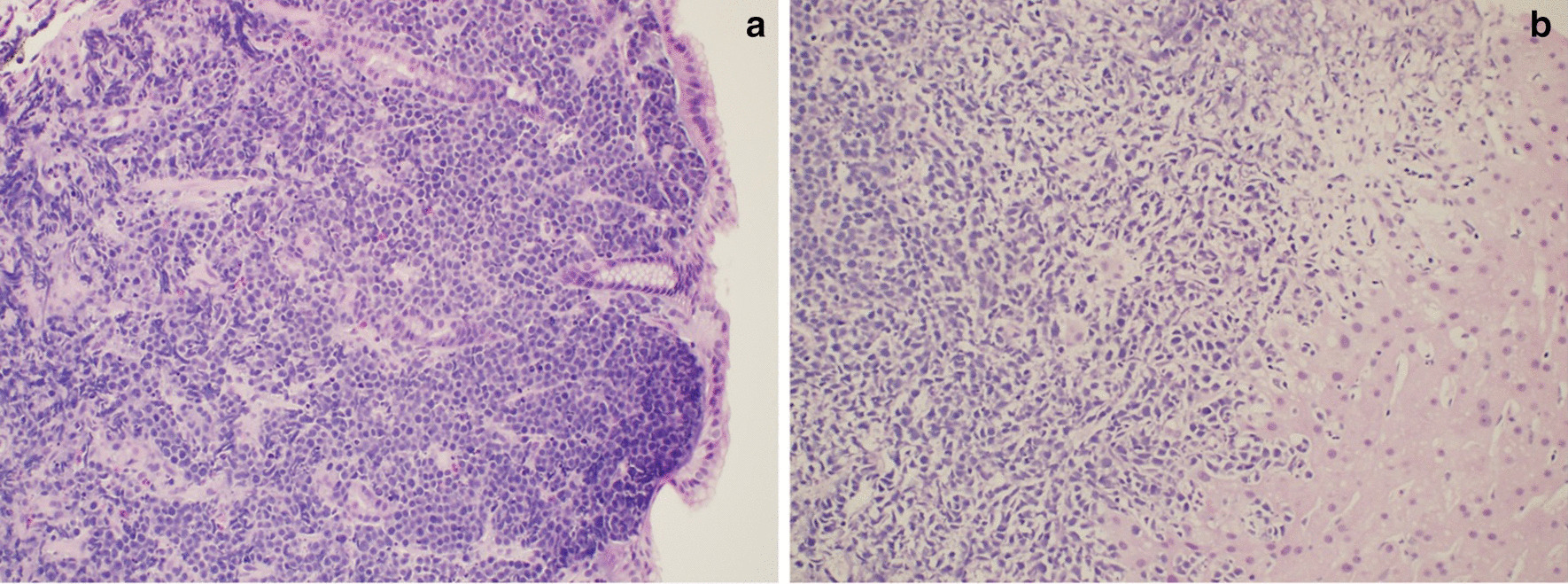
**a** Stomach biopsy demostrates high grade B-cell lymphoma invading the lamina propria of the stomach. **b** Liver biopsy demonstrates large sheets of atypical cells similar in morphology to gastric biopsy

**Fig. 4:**
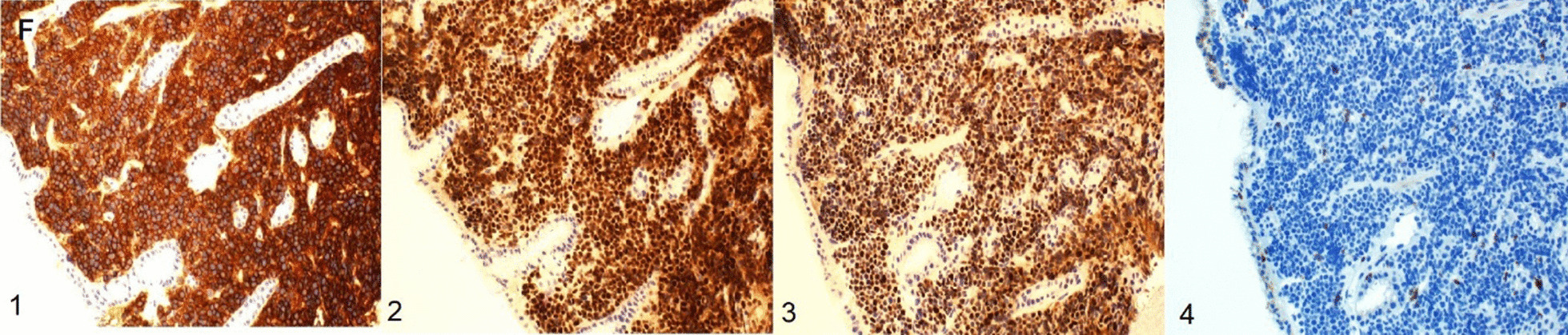
Immunostaining positive for diffssuse B cell lymphoma, (1) CD20, b cell marker; (2) Pax5 b cell marker; (3) BCL6; (4) CD3 T cell marker

A 55-year-old male with a past medical history of anemia and long-standing alcohol abuse with recent diagnosis of cirrhosis (Child’s Pugh Score B, MELD-Na 22) decompensated by portal hypertension in form of abdominal ascites, and medium-size esophageal varices, presented with 2 weeks of worsening epigastric abdominal pain and distention. The patient drinks six cans of beer daily for 10 years. Denies smoking or eliciting recreational drugs. No prior surgical intervention. On physical examination, the patient looks cachectic. He has minimal scleral icterus and trace lower extremities edema. Abdominal examination notable for a mild epigastric tenderness and abdominal distention with shifting dullness. His laboratory data demonstrates complete blood count (CBC) with WBC 8. 7 K/MCL, hemoglobin (Hb) 9.1 gm/dl, Hct 26.8%, and platelets 46 k/MCL. The coagulation panel demonstrates an INR of 2.0. Abnormal liver chemistry, alanine transaminase (ALT) of 44 IU/ml, aspartate aminotransferase (AST) of 67 IU/ml, alkaline phosphatase (ALP) of 253 mg/dl, and a total bilirubin 5.2 mg/dl (direct bilirubin 3.8). The work-ups for abnormal liver chemistry were unremarkable including viral hepatitis A, B and C. Abdominal triple-phase CT was noteworthy for cirrhosis, mild to moderate ascites, and multiple new hypodense lesions throughout the liver, the largest being a 6 cm in diameter in the right lobe. The lesions were classified as an intermediate probability for HCC based on the LI-RADS classification system (LI RADS 3) (Fig. 1a, b). The tumor marker for AFP was normal. Three months before admission the patient had an unremarkable upper endoscopy (EGD) and colonoscopy for an indication of iron deficiency anemia (IDA) which demonstrates no obvious luminal lesion. The hospital course was complicated by upper gastrointestinal bleeding with hematemesis required blood transfusion. Diagnostic EGD showed large non-bleeding esophageal varices, severe portal hypertensive gastropathy (PHG), and multiple new umbilicated, ulcerated, firm, submucosal nodules throughout the gastric body (Fig. 2). Targeted liver biopsy performed given the suspicious radiological liver features for HCC in the background of cirrhosis Biopsy of the submucosal gastric nodules revealed high-grade B-cell lymphoma involving the lamina propria, without evidence of Helicobacter Pylori. Neoplastic cells were positive for CD20, Pax-5, BCL-6, CD10, BCL-2, and negative for CD3. Ki67 index was 95% positive for lymphoma cells (Figs. 3a, b, 4). Further, tumor markers including carcinoembryonic antigen (CEA) and carbohydrate antigen 19-9 which were normal. The cell morphology and immunostaining profile of the liver biopsy was identical to the gastric submucosal nodules, thus confirming the diagnosis of primary gastric DLBCL with metastasis to the liver (Figs. 3b, 4). The patient received one session of chemotherapy with low-dose Rituximab-Cyclophosphamide, Doxorubicin, Vincristine, and Prednisone (R-CHOP) knowing that lymphoma is chemosenstive tumor and patient continues to have stable bilirubin since hospital admission (MELD-Na 21). Unfortunately, his bilirubin levels started to rise up to 25 mg/dl over 3 weeks period. His hospital course was complicated with septic shock secondary to SBP and disseminated intravascular coagulation (DIC) resulting in his death.

## Discussion and conclusions

Primary gastric lymphoma constitutes < 5% of all primary gastric neoplasms. The GI tract accounts for 30–40% of all extra nodal non-Hodgkin’s lymphomas, making it the most common extra-nodal site [2]. Primary gastric lymphoma encompasses a spectrum of diseases ranging from indolent low-grade marginal lymphoma to mucosa-associated lymphoid tissue (MALT) lymphomas, to aggressive diffuse B cell lymphomas (DBCL) [2].

The incidence of large B cell lymphoma in the general population is 5–7 cases per 100,000 cases per year while the incidence of DBCL in cirrhosis is extremely rare with no clear actual incidence in literature [3, 4]. It has been postulated in previous studies that dysregulation in immune function contributes to the disease process. Specifically, chronic antigen stimulation contributes to the development of lymphoproliferative disorders leading to monoclonal gammopathy. Viral hepatitis may also be a source for antigen stimulation in the case of Hepatitis B [5]. Similarly, H. pylori is also thought to cause antigenic stimulation that can lead to monoclonal gammopathy and eventually to malignant lymphoproliferative disorder. Platin based chemotherapy has been described as a risk factor for lymphoma in cirrhotic patients treated for HCC [6]. To our knowledge, there is no clear correlation between lymphoproliferative disorder such lymphoma and cirrhosis in the absence of antigenic stimulation (viral hepatitis B & C).

The clinical presentation of PGL is nonspecific, however, abdominal pain and weight loss are the most common manifestations. Gastrointestinal bleeding due to lymphoma cells infiltrating into the stomach can occur and, in some instances, may lead to perforation [7, 8]. A more dramatic presentation can be seen in patients with decompensated cirrhosis especially with underlying coagulopathy from chronic liver disease. According to the Lugano classification of DBCL, isolated stomach involvement denotes localized disease. Distant metastasis with advanced lymphoma involves the liver as in our case causing multiple hepatic lesion resembling metastatic HCC. These hepatic lesions lack the radiologic characteristic of HCC such as arterial enhancement, pseudo capsule, and washout further supporting lymphoma as the etiology for the clinical findings.

Diagnostic imaging with a cross-sectional CT scan is necessary to stage disease [9]. A positron emission tomography (PET) scan is helpful as it can demonstrate an uptake in the stomach and the liver to express tumor activity but it won’t provide tissue diagnosis to differentiate synchronous versus metachronous tumor. Endoscopic ultrasound may play a role in early disease (stage I, II) [10]. The treatment of PGL includes chemotherapy, surgical resection, and some instances radiation therapy. Anti-CD-20 monoclonal antibody with Rituximab in combination with CHOP therapy is a mainstay treatment approach for patients with lymphoma. R-CHOP made DBCL a curable disease with a good prognosis with a five to ten-year survival rate approaching 55% [9]. Surgical resection is curative especially for localized disease [2, 9]. Certain considerations are crucial for such patients with cirrhosis such as MELD-Na score prior to considering chemotherapy treatment and evaluating patient’s surgical candidacy according to the Child’s Pugh A classification. The overall disease prognosis, five to ten-year mortality rate for PGL stage I, 87% and stage II, 60% [11]. The 3 year survival rate for stage III & IV is 43–65% [9, 11]. Prognosis expect to be lower in the presence of cirrhosis but there is no clear literature evidence as of yet.

In conclusion, we report a case of PGL with metastasis to the liver in a patient with decompensated cirrhosis. This case depicts the diagnostic uncertainty of recognizing metastatic PGL in patients with underlying cirrhosis. It highlights the importance of targeted liver biopsies in patients with cirrhosis with liver lesions and normal AFP values.

## Data Availability

Not applicable.

## Abbreviations

PGL: Primary Gastric lymphoma
ENHL: Extra-nodal Non-Hodgkin lymphomas
IBD: Inflammatory bowel disease
SLE: Systemic lupus erythematosus
HTLV: Human T-lymphotropic virus
HCC: Hepatocellular cancer
AFP: Alpha-feto-protein
CEA: Carcinoembryonic antigen
ALT: Alanine transaminase
AST: Aspartate aminotransferase
ALP: Alkaline phosphatase
PHG: Portal hypertensive gastropathy
MALT: Mucosa-associated lymphoid tissue lymphoma
DBCL: Diffuse B cell lymphoma
EUCAST: European Committee on Antimicrobial Susceptibility Testing
R-CHOP: Rituximab-Cyclophosphamide, Doxorubicin, Hydrochloride, Hydroxy daunomycin and Vincristine Sulfate
